# Decision-making in social contexts in youth with ADHD

**DOI:** 10.1007/s00787-016-0895-5

**Published:** 2016-08-23

**Authors:** Ili Ma, Nanda N. J. Lambregts-Rommelse, Jan K. Buitelaar, Antonius H. N. Cillessen, Anouk P. J. Scheres

**Affiliations:** 1 Behavioural Science Institute, Radboud University, Nijmegen, The Netherlands; 2 Donders Institute for Brain, Cognition and Behavior, Radboud University, Nijmegen, The Netherlands; 3 Radboud University Medical Center, Nijmegen, The Netherlands

**Keywords:** ADHD, Motivation, Decision-making, Social

## Abstract

This study examined reward-related decision-making in children and adolescents with ADHD in a social context, using economic games. We furthermore examined the role of individual differences in reward-related decision-making, specifically, the roles of reward sensitivity and prosocial skills. Children and adolescents (9–17 years) with ADHD-combined subtype (*n* = 29; 20 boys) and healthy controls (*n* = 38; 20 boys) completed the ultimatum game and dictator game as measures of reward-related decision-making in social contexts. Prosocial skills were measured with the Interpersonal Reactivity Index. The ADHD group had a larger discrepancy between ultimatum game and dictator game offers than controls, indicating strategic rather than fairness driven decisions. This finding was supported by self-reports showing fewer individuals with ADHD than controls who considered fairness as motive for the decisions. Perspective taking or empathic concern did not differ between groups and was not significantly associated with offers. In conclusion, the results suggest that rather than a failure to understand the perspective of others, children and adolescents with ADHD were less motivated by fairness than controls in simple social situations. Results encourage the use of economic games in ADHD research.

## Introduction

Attention-deficit/hyperactivity disorder (ADHD) is a common childhood-onset disorder characterized by age-inappropriate levels of inattention and/or hyperactivity–impulsivity that are present in multiple settings [3, 4]. In addition to its core symptoms, ADHD is associated with an increased risk for behaviors reflecting poor decision-making skills [54] such as unsafe driving [54], sustaining injuries [13] and social dysfunctions [47].

Two widely studied factors contributing to decision-making that are part of influential theoretical accounts of ADHD are alterations in cognitive control e.g., [5], [9], [53] and reward processing [38]. In line with growing awareness of ADHD heterogeneity e.g., [9], [60], decision-making in the context of ADHD may be best viewed as the result of *interactions between* cognitive control and motivational states, e.g., [8, 61]. This relates to decision-making contexts in which the motivational significance of stimuli needs to be appraised, also referred to as reward-related decision-making, when the motivational component pertains to monetary outcome, e.g., [36, 43, 68].

Research on reward-related decision-making in individuals with ADHD has mainly focused on risk tasks (gambling) and temporal discounting tasks (choice between small immediate and large delayed rewards). Children and adolescents with ADHD show increased risky performance and a stronger preference for small immediate rewards compared with controls [21, 32].

These two lines of research provided important insight into reward-related decision-making in individuals with ADHD. However, this research was conducted using non-social tasks. Importantly, social and emotional impairments are associated with ADHD [67]. Therefore, it is relevant to study decision-making while appraising the motivational significance of stimuli in *social interactions* in individuals with ADHD. Indeed, children with ADHD demonstrate fewer prosocial behaviors such as empathy (especially those with comorbid conduct problems [29], have less stable friendships [48] and show more peer rejection than children without ADHD [15, 30, 31, 42, 52, 65], but also see [19]. Peer rejection and social dysfunction are important negative consequences of ADHD and are associated with poor long-term outcomes including cigarette smoking, delinquency, anxiety and global impairment, emphasizing the relevance of investigating social behavior in children with ADHD [44, 47]. Adding social contexts to reward-related decision-making tasks will thus increase the ecological validity. Also, it provides insight into interactions between reward-related decision-making and social functioning.

Paradigms assessing *social* reward-related decision-making have been widely used in the field of economics. One of its aims is identifying factors that influence financial decision-making. Such paradigms, called *economic games*, have been extensively investigated and their typical effects are well documented. One such game, the ultimatum game (UG), assesses ultimatum bargaining [24]. An ultimatum is defined as a situation in which one player (Proposer) proposes a division, which the second player (Responder) can either accept or reject. The Responder can either accept the offer; both players then receive the money (divided as proposed). Alternatively, the Responder rejects, and both players will receive nothing. Always accepting the offer would be most economically beneficial (small monetary gain is better than none), but it has been well established that relative offers of <20 % are typically rejected [24]. This demonstrates that people do not exclusively value material gain [7, 25]. In the current study, participants take on the Proposer role. Therefore, Proposers have to consider that a too low offer may be rejected, whereas a reasonably equal split has a high acceptance chance. Accordingly, offers made in the UG are typically close to 50 % of the total [6].

In addition to these strategic considerations, another possible motive for proposing equal splits in the UG is fairness [6, 18, 55, 67]. The dictator game (DG) can be used in addition to the UG to tease apart strategy from fairness. This paradigm is identical to the UG except for the crucial fact that the Responder cannot reject. Thus, both players will receive the Proposers’ offer, regardless of the Responders’ opinion [33]. Therefore, in the DG, fair offers are likely motivated by fairness, and not strategy, e.g., [6, 40]. Accordingly, offers in the DG are typically lower than in the UG [6]. If the discrepancy between offers in both games is high (e.g., the participant offers 50 % of their money in the UG but 0 % in the DG), then the UG offer reflects strategic bargaining. In other words, the player increased the acceptance likelihood in the UG by offering a share that will not likely be rejected [55]. Alternatively, if the discrepancy between both games is low (e.g., when the Proposer offers 50 % in both the UG and DG games), then the UG offer likely reflects a fairness motive, because in the DG the Responder is not able to reject the offer. Thus, the combination of games can be used to assess motives behind reward-related decision-making.

These games have been thoroughly studied in children and adolescents without ADHD, for review see [12]. In 3- to 8-year-olds, preferences shift from more selfish DG decisions to relatively more equal splits with increasing age [16]. Children at age 8–9 years no longer differ from adolescents and adults in their DG decisions [27]. Strategic social decision-making follows a more prolonged development. Six to 13-year-olds show stable DG offers but not UG offers, and thereby the difference between the offers increases with age. Higher UG offers were positively associated with individual differences in response inhibition and left dorsolateral prefrontal cortex (DLPFC) response and cortical thickness [61]. These games have been well studied and both show robust effects of social context (UG versus DG) in healthy populations. In addition, the instructions are easy to understand. Straightforward instructions and short tasks are preferred when comparing individuals with psychopathology such as ADHD with healthy controls, because of the possibly impaired working memory or attention deficits in the psychopathology group. Therefore, these paradigms are suitable for studying social decision-making in children with and without ADHD but also across different age groups. The first is especially an understudied, but relevant area of research, also see [58].

In summary, the primary goal of this study was to assess reward-related decision-making in social contexts in children and adolescents with ADHD and controls. Specifically, considerations of fairness versus strategic bargaining were assessed by administering the UG and DG in the role of the Proposer. Self-reports provided more insight into the motives behind decisions. Because ADHD is associated with atypical reward sensitivity and lower cognitive control [26, 38], the main hypothesis was that the ADHD group would aim to maximize their monetary gain. If participants with ADHD would make low offers in both games relative to controls, this would be interpreted as a limited consideration of fairness and others’ perspective. On the other hand, if individuals with ADHD would make lower offers than controls in the DG but not in the UG (large discrepancy), this would indicate strategic bargaining, while also understanding others’ perspective. As a secondary goal, group differences in prosocial behavior were examined, also in relation to offers. We expected prosocial behavior to be associated with fairness preference.

## Methods

### Participants and screening procedure

The final sample consisted of 67 children and adolescents in the age range of 9–17 years. Participants with ADHD-combined subtype (ADHD-C; *n* = 29) were recruited through Karakter, Child and Adolescent Psychiatry Department of the Radboud University Nijmegen Medical Centre. Control subjects (*n* = 38) were recruited through local advertisements and schools. The groups did not significantly differ on gender, age, and IQ (see Table 1).

**Table 1:** Participant characteristics

Variable	Controls (*n* = 38; 20 boys)	ADHD (*n* = 29; 20 boys)	Group difference
	Mean, SD	Mean, SD	
Age	13.24 ± 2.32	12.31 ± 2.38	*p* = .11
Estimated IQ	107.55 ± 14.47	101.62 ± 12.03	*p* = .08
DBDRS (parents)^a^			
Inattention	10.44 ± .93	15.18 ± 1.60	*p* < .001
Hyperactivity/impulsivity	10.38 ± 1.02	14.48 ± 2.09	*p* < .001
ODD	10.82 ± 1.38	12.94 ± 1.62	*p* < .001
CD	11.44 ± 1.46	12.39 ± 1.85	*p* = .03
CBCL (*T* scores)^b^			
Social problems	51.43 ± 2.44	59.16 ± 7.69	*p* < .001
Rule-breaking behavior	52.06 ± 3.55	57.96 ± 7.54	*p* = .001
Aggressive behavior	50.69 ± 1.94	62.96 ± 7.33	*p* < .001
CBCL DSM scales (*T* scores)			
Affective problems	52.63 ± 3.08	61.88 ± 7.16	*p* < .001
ADHD	51.37 ± 3.20	70.84 ± 5.89	*p* < .001
ODD	51.43 ± 3.15	61.12 ± 8.69	*p* < .001
CD	51.31 ± 2.61	58.88 ± 6.41	*p* < .001
IRI^c^			
Perspective taking	2.1 ± 0.72	2.08 ± 0.69	*p* = .78
Empathic concern	2.25 ± 0.49	2.11 ± 0.70	*p* = .36

^a^Disruptive Behavior Disorder Rating Scale. Standardized scores. For the norm group, the average score is 10 ± 3. ^b^ Child behaviour checklist. ^c^ Interpersonal Reactivity Index. *α* was set at .05

Clinical assessment: All ADHD subjects were previously diagnosed with ADHD-C by accredited clinical psychologists/psychiatrists. Furthermore, the diagnosis of ADHD-C was re-confirmed by a trained psychologist at the time of the study using a structured parent interview: the Diagnostic Interview Schedule for Children (DISC–IV) [57]. Participants with ADHD were excluded if they met the psychiatric disorder criteria other than ADHD on the DISC-IV, except for oppositional defiant disorder (ODD). Because of the high comorbidity with ADHD, subjects with comorbid ODD (*n* = 9) were included. Additional questionnaires were used as descriptive (not diagnostic) instruments (Table 1). Participants with ADHD who were using psychostimulants (*n* = 20) discontinued their medication 24 h prior to testing [20].

Controls were excluded if they met criteria for psychiatric disorders on the DISC-IV, as assessed by a trained psychologist, or scored within clinical range on the Disruptive Behavior Disorder Rating Scale (DBDRS; [50] or CBCL (CBCL; [1]. Participants in both groups were required to have an estimated IQ >75 based on the vocabulary and block design of the Dutch Wechsler Intelligence Scale for Children (WISC-III; [34].

### Interpersonal Reactivity Index (IRI)

The IRI was included to measure the prosocial behaviors, perspective taking and empathy. The 28-item IRI self-report is designed for the assessment of empathy [14], defined as “the reactions of one individual to the observed experiences of another (p. 1)”. Participants responded on a five-point Likert scale. The IRI consists of four subscales: perspective taking, empathic concern, fantasy and personal distress with high reliability (Chronbach’s alpha .72, .70, .78 and .73, respectively, for early adolescents [28]. We used the subscales perspective taking and empathic concern. Perspective taking refers to the ability to spontaneously adopt the psychological viewpoint of someone else. Empathic concern addresses feelings of sympathy and concerns for unfortunate others [14]. In the current sample, the reliability of perspective taking and empathic concern (subscales of interest) were good (Chronbach’s alpha .74 and .68, respectively).

### Reward-related social decision-making tasks

#### Ultimatum game

Participants played a one-shot UG in the role of the Proposer. They were informed that they were randomly selected to fulfill the role of the Proposer and instructed to distribute €5 between themselves and an anonymous partner (Responder). They were told that the Responder was another participant in the study of the same age who could either accept or reject their offer. If the offer was accepted, the money would be divided as proposed. If the offer was rejected, neither player would receive anything. It was emphasized that the game was played for real money and that they would be paid on their next visit (study procedure) based on the decision made by the Responder. It was further emphasized that all players would remain anonymous and that they would not meet or interact in any of the subsequent experiments. The participants indicated their offer on a paper with two rows indicating amounts displayed in 50-cent increments from €0 to €5. In reality, there was no responder and when offers fell below €2 the experimenters told participants during their next visit that the offer was rejected. If the offer was higher or equal to €2, participants were told that their offer was accepted and they were paid accordingly. Rejecting offers that are much lower than 50 % of the total amount would be a realistic reaction [49].

#### Dictator game

Participants played a one-shot DG, also in the role of the Proposer. The procedure was the same as outlined above, except that the Responder did not have the option to reject the offer and participants were explicitly informed of this. Furthermore, they were told that they would make this offer to another, anonymous partner. After their choice, they received the amount that they wanted to keep for themselves immediately. They were not informed of this immediate payoff before their choice.

#### Self-reports

After completing both games, all participants were asked to respond to three questions concerning the games: “How did you make your decision during the game?” (for UG and DG) and “How difficult was it to make these decisions?” These were asked as open-ended paper and pencil questions to avoid evoking response biases. The responses were evaluated qualitatively and categorized based on the content (see below).

#### Study procedure

This study was approved by the local medical ethics committee consistent with the Dutch Act on Medical Research Involving Human Subjects. Informed consent was obtained from all participants and their parents. This study consisted of three consecutive test days. This experiment was part of a larger project with other experimental studies reported elsewhere. Participants completed the IQ assessment, UG and DG, and questionnaires in the first session in the behavioral laboratories of the Radboud University. The families received €30 for participating in all the sessions.

### Analyses

#### ADHD versus control comparisons for offers

The dependent variables were not normally distributed. Therefore, we performed non-parametric Mann–Whitney *U* tests to examine whether participants with ADHD differed from controls on UG offers, DG offers and strategic bargaining (UG–DG offers). Effect sizes for the Mann–Whitney *U* (denoted as *r)* are computed by dividing the Z statistic by the square root of the sample size, with small, medium and large effect sizes of *r* = .01, 0.3 and 0.5, respectively [17]. Significant Mann–Whitney U tests were followed by categorizing DG offers into fair (50 %), intermediate (0 > 50 %) and unfair (0 %) decisions. Groups where then compared on categories using a Chi-square test and a significant result was further examined by converting the adjusted residuals (Z-scores) to Chi-square values and testing those against a Chi-square distribution (Bonferroni corrected *α* = .0167) as recommended to identify group differences per category following significant Chi-square tests [64]. Age did not correlate with UG offers (*rho* = .01, *p* = .96) or DG offers (*rho* = .08, *p* = .50). Therefore, it was not included as a factor in the analyses.

#### Self-reports

To examine between-group differences in fairness versus strategic considerations in the UG and DG offers and their difference score, responses were stratified into three categories and compared between diagnostic groups using Bonferroni-corrected Chi-square tests (*α* = .0167). Responses on the UG and the DG question could be stratified into the categories: (1) fair or (2) strategic. Responses to question 3 were categorized into two options: (1) difficult or (2) easy. For all three questions, the answer was assigned to a third category if a participant responded not to know their motive. To assess which consideration contributed to the offers in the games, a Kruskal–Wallis test was conducted for the three response categories. If the Kruskal–Wallis test was significant, post hoc Bonferroni-corrected Mann–Whitney *U* tests were conducted (*α* = .0167). For these analyses, the coded responses (fair/strategic/do not know) to the self-report questions were entered as independent variables, while offer sizes and the difference (UG–DG offers) were the dependent variables.

#### Reward-related decision-making associations with IRI

To examine whether empathic concern and perspective taking correlated with strategic bargaining, Spearman rho correlations were computed across groups. Bonferroni corrections for multiple comparisons were applied, *α* = .006. Ten participants (7 controls, 3 ADHD) did not complete the scales and were excluded.

## Results

### Descriptive statistics

In accordance with previous reports [6], the mean offer made in the UG was between 40 and 50 % of the total: 48 % (*M* = €2.40; SD = 0.26). The mode was €2.50 and the range €1–3. As expected [6], the current study shows that the mean offer in the DG was lower than that in the UG and close to 20 % of the total: 19 % (*M* = €0.90; SD = 1.05). The mode was €0 and the range €0-2.50.

For the IRI, the mean score over all participants for perspective taking was 12.67 (SD = 4.18); for empathic concern, it was 13.14 (SD = 3.52). There were no significant group differences in either of the scales (Table 1).

The ADHD and control groups differed in the DBDRS and CBCL scales (Table 1). The ADHD and control groups did not significantly differ in age, IQ or gender (*χ* ^*2*^(1,75) = 1.74, *p* = .24; Table 1).

### ADHD versus control comparison for offers

**Fig. 1:**
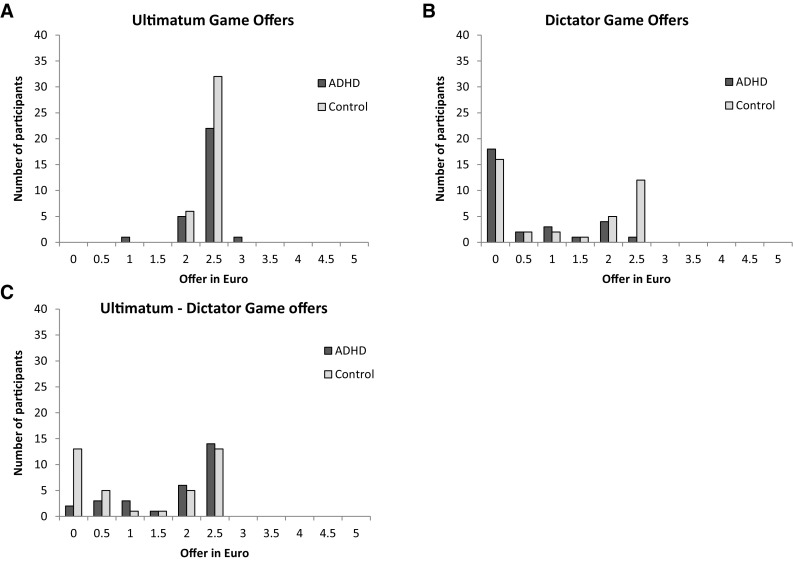
Histograms show the number of participants per decided offer for each group. **a** Number of participants for each ultimatum game offer per group. **b** Number of participants for each dictator game offer per group. **c** Number of participants for each difference score between ultimatum and dictator game offers per group

For UG offers, we found no significant difference between the control and ADHD group (Fig. 1a; *U* = 537.00, *p* = .80, *r* = −.03). In the DG, the ADHD group made lower offers than controls (Fig. 1b; *U* = 381.50, *p* = .02, *r* = −.29). The Chi-square analysis clarified this finding by showing a group difference in the fair/intermediate/unfair categories (*χ* ^*2*^(2,67) = 8.37, *p* = .02). This group difference was driven by the lower amount of fair offers from ADHD participants (50 % of the total amount) (*n* = 1) than controls (*n* = 12; *Z* = ±2.90, *p* = .004). There were no group differences in the intermediate category (*Z* = ±.70, *p* = .48) or in the unfair category (*Z* = ±1.60, *p* = .11).

As for strategic bargaining, the ADHD group (median (Mdn) = 2.00, range = 0–2.50) had a significantly larger difference between the UG and DG offer than the control group (Mdn = 1.25, range = 0–2.50, *U* = 391.50, *p* = .04, *r* = .26), indicating that the ADHD group showed more strategic bargaining than controls (Fig. 1c).

Importantly, the effects remained the same when excluding individuals with comorbid ODD (*n* = 9; there were no participants who met criteria for comorbid CD or other comorbidities): Children with ADHD made lower offers on the DG compared with controls (*U* = 258.50, *p* = .03, *r* = −.28) and there was no significant group difference in the UG (*U* = 361.00, *p* = .57, *r* = −.06). Furthermore, there were no significant correlations between ODD/CD symptoms on either the DBDRS or CBCL and offers made on both games (for DG all *p* > .10; for UG all *p* > .07). There were also no significant correlations between offers on either game and the CBCL scales social problems, rule-breaking behavior or aggressive behavior (for DG all *p* > .40; for UG all *p* > .17). This suggests that our effects were not driven by the individuals with comorbid ODD or CD symptoms.

### Self-reports

For Question 1 (decision UG), the ADHD and control group did not significantly differ in the number of participants per response category (*χ* ^*2*^(1,67) = .48, *p* = .62). The UG offer did not significantly differ per response category (*χ* ^*2*^(1,67) = 3.06, *p* = .11). The mean ranks were 36.65 for fairness and 30.93 for strategy. No participants responded with “do not know”.

For Question 2 (decision DG), the overall Chi-square test was significant (*χ* ^*2*^(2, 67) = 9.73, *p* = .006): fewer participants with ADHD (20.7 %) than controls (50 %) reported fairness (*Z* = ±2.50, *p* = .01). There was no group difference in strategy considerations (controls 50 %, ADHD 65.5 %, *Z* = 1.69, *p* = .19) or in the category “do not know” (ADHD 13.8 %, controls 0 %, *Z* = 5.76, *p* = .02, *α* = .017). There was a difference between self-reports and offers (*χ* ^*2*^(2,67) = 41.75, *p* < .001): participants who answered with fairness made higher DG offers than those who reported strategy (*U* = 228.00, *p* < .001, *r* = .78), but not those who responded with “do not know” (*U* = 24.00, *p* = .11, *r* = .20). Those who considered strategy did not differ from those who responded with “do not know” (*U* = 76.00, *p* < .99, *r* = .34). Mean ranks were 51.68 fairness, 21.78 strategy and 39.63 “do not know”.

For Question 3 (difficulty deciding), the ADHD and control group did not differ (*χ* ^*2*^(2,67) = .25, *p* = .30). Kruskal–Wallis tests showed that offers in the UG, DG and the difference between UG and DG offers were unrelated to self-reported difficulty (UG (*χ* ^*2*^(2,67) = 1.16, *p* = .49), DG (*χ* ^*2*^(2,67) = .11, *p* = .95), difference (*χ* ^*2*^(2,67) = .08, *p* = .97). The UG mean ranks were 35.27 for fairness, 32.43 for strategy and 28.67 for “do not know”. The DG mean ranks were 33.44 for fairness, 34.96 for strategy and 34.33 for “do not know”.

### Individual differences: IRI scales

There were no correlations between perspective taking (*rho* = −.11, *p* = .40) or empathic concern (*rho* = −0.28, *p* = .03, *α* = .006) with DG offers.

## Discussion

This study investigated reward-related decision-making in social contexts in children and adolescents with ADHD compared with controls by employing the well-established ultimatum game UG [24] and dictator game DG [33]. By investigating the discrepancy in offers between both games, we were able to disentangle strategic from fair decisions [55]. In addition, self-reports gave more insight into the reasons for the offers. As a secondary objective, the associations between strategic bargaining and empathy/perspective taking were examined. The main hypothesis was confirmed: those with ADHD maximized their gains more than controls by lowering their DG offers as compared to UG offers more than controls. This indicated increased strategic bargaining in the ADHD group. Results for these objective measures were consistent with self-reports; the ADHD group showed less fairness considerations in the DG than controls. Finally, in contrast to expectations, no relationship between offers and prosocial skills was found.

The ADHD group showed more strategic bargaining, as reflected by lower DG offers relative to UG offers, whereas in comparison the control group showed less discrepancy between the UG and DG offers. This higher discrepancy between UG and DG offers in the ADHD group indicates that they aimed to maximize the amount of their monetary gain [55]. Self-reports confirmed that significantly fewer individuals in the ADHD group considered fairness as a motive for DG choices. This indicated that the ADHD group aimed to maximize the likelihood and the amount of their monetary gain [55]. The finding that offers in the UG approximated 50 %, corresponds with a high likelihood of acceptance and concurs with previous findings using this paradigm with anonymous players [24]. This confirms that all participants understood the UG status quo: potential rejection if the Responder feels being treated unfairly, resulting in zero payoff. It is thereby implied that the group differences in strategic bargaining were not likely due to severe perspective-taking deficits. Others have shown that young children who had not yet developed an understanding of other’s perspective made lower UG offers than those who had developed such an understanding [63]. A lack of perspective-taking ability is therefore associated with low UG offers that have a high probability of being rejected. Our data imply that such a lack of understanding was not present in our sample, further supported by the fact that there were no group differences on the IRI perspective-taking scale. Finally, it is important to note that the level of complete selfishness (DG offers of 0) was comparable between groups. The group difference arose specifically in the DG, where participants with ADHD made lower offers than controls, thereby maximizing their own gains to a larger extent than controls. This resulted in a larger difference between the UG and DG in the ADHD group compared with controls. Our findings suggest that the ADHD groups’ decisions were more driven by gain maximizing motivations than social motivations compared with the control group.

The finding of more strategic bargaining by the ADHD group is in line with the notion of altered reward sensitivity in ADHD [26]. However, altered reward sensitivity is not the only factor at play. A different study, which focused on age effects in strategic bargaining, demonstrated that an age-related increase in strategic bargaining was associated with a developmental increase in self-control [61]. Assuming that individuals with ADHD in this study would have a relatively weak self-control, they would be expected to demonstrate less rather than more strategic bargaining than controls. It needs to be noted, however, that the link between strategic bargaining and high self-control in the study by Steinbeis was driven by correlations between UG offers and self-control, and not by DG offers and self-control. In the current study, the link between strategic bargaining and ADHD-C was driven by the DG offers, and not UG offers. Despite this difference, the fact that the ADHD group actually showed more strategic bargaining, might suggest that the monetary gain motivated the ADHD group more than controls to recruit more self-control. Although the role of self-control in the current finding is speculative until future research is conducted, prior studies have shown that monetary rewards can normalize self-control (response inhibition) in youth with ADHD to the level of controls, see Ma et al. [39] for a meta-analysis. Clearly, future research is needed in which the role of self-control and reward sensitivity/motivation in strategic bargaining in those with ADHD is directly assessed by including measures of self-control (such as the stop task) and reward sensitivity.

The current study found that the strategy in the ADHD group was driven by the lower number of individuals making fair DG offers (50 %), but *not* a higher number of participants making unfair (0 %) DG offers. This finding may be understood with the model by Myrseth and Fishbach [46]. According to this model, conflict arises in DG, consisting of a choice between being fair or egoistic. Not identifying conflict leads to indulging (keeping all the money). Identification of conflict leads to either restraint (allocate money to the Responder) or failed restraint (indulging). The outcome of the conflict is shown to depend on trait self-control [40, 41]. Although empirical evidence is not entirely consistent regarding the cognitive control and DG fairness relation [56], egoistic DG decisions are inherently little reliant on cognitive control as there is no risk of rejection. Selfish tendencies do not need to be overruled, as there is no consequence to being selfish. In our study, a lack of detecting conflict in the ADHD group does not explain the group difference; groups did not differ in €0 DG offers. This further supports the suggestion that for individuals with ADHD, fairness may not be a sufficient motivator to recruit cognitive control to override selfish tendencies.

The current study did not find convincing age effects, even irrespective of diagnosis. In the DG, this was to be expected, as prior studies have not found clear differences in fairness between children and adolescents of similar age as those included in our study [22] and younger ages [16]. For the UG, previous research is less conclusive [22, 27, 45, 62]. For example, [45] found that children’s offers decreased with age, while Harbaugh et al. [27] found the opposite result, and Gummerum et al. [22] found no age effects, possibly due to design differences. However, developmental differences may arise more clearly in somewhat more complicated contexts where intentionality or individual effort should be balanced against strict equality preferences [2, 23]. Children in the role of Responder have been shown to increasingly incorporate information regarding others’ intentions in their decisions with age [23, 62]. For example, it occurs more frequently with increasing age that a moderately unfair offer is accepted when the only alternative choice for the Proposer would have been to choose an even more unfair offer [62]. Similar effects have been shown for individual achievement considerations [2]. Future research may aim to further investigate the developmental trajectory of decision-making in individuals with ADHD, using a combination of games that systematically manipulate the contextual factors that affect the decisions. In addition, studying a wider age range is likely to show stronger developmental effects, especially the inclusion of younger children [16, 22].

The current study had a number of strengths and limitations. Strengths included the use of solid task designs, as the UG and DG are well-established paradigms to assess decision-making in a social context. Applying these to ADHD is an innovative approach to experimentally study reward-based decision-making in social contexts. Furthermore, by combining the UG and DG, we were able to disentangle strategic bargaining from fairness motives. Limitations of this study include the lack of inclusion of self-control and reward sensitivity measures. Therefore, future research may expand the scope of the current study. First, although the literature has supported the suggestion that cognitive control is involved in these tasks, future ADHD studies may directly examine the involvement of cognitive control by including tasks such as the Stop Signal Task [37]. Additionally, including functional brain imaging techniques to study associated activation in relevant brain regions such as the dorsolateral prefrontal cortex will be of interest. Second, the study was not designed to extensively measure social functioning in daily life. Future studies can address this by relating the experimental findings to, for example, questionnaires and/or sociometric data [10]. In addition, social decision-making clearly is influenced by perceptions of interpersonal closeness (e.g., [11]. Children and adolescents with ADHD experience social difficulties with people whom they frequently interact with (i.e., teachers, parents and/or siblings). Therefore, an important future step related to ecological validity would be to examine whether children with ADHD modulate their decision-making based on feedback from another person such as a teacher to maintain or develop positive relationships. Third, youth with ADHD demonstrate a positive illusory bias regarding self-perceptions and social competence [35, 51]. Although this does not confound the main task findings, future studies should include social competence measures from parents. Finally, a focus on the Responder role will provide insight into participants’ emotional and potentially aggressive reactions to experienced unfairness. Future research is required to uncover how individuals with ADHD will behave when engaged as Responders in these economic games. It can be hypothesized that children and adolescents with ADHD will show a higher rejection rate due to emotion regulation impairment and reactive aggression [59, 67].

## Conclusion

This study investigated social reward-related decision-making in children and adolescents with ADHD using the single shot dictator game (DG) and ultimatum game (UG) in the role of the Proposer. In the DG, the likelihood of monetary payoff does not require considering the perspective of others, and children and adolescents with ADHD made less fair offers than controls. In the UG, which does require considering the perspective of another, there was no group difference. Both the ADHD and control groups made offers that were likely to result in a monetary payoff. This pattern illustrates that the ADHD group was characterized by increased strategic bargaining. The results suggest that rather than a failure to understand the perspective of others, children and adolescents with ADHD are less motivated by fairness and more by monetary gain than controls in simple social situations.
